# Public attitudes towards dialects: Evidence from 31 Chinese provinces

**DOI:** 10.1371/journal.pone.0292852

**Published:** 2023-10-12

**Authors:** Tianxin Li, Xigang Ke, Jin Li

**Affiliations:** 1 Department of Literature, Shaanxi Normal University, Xi’an, Shaanxi, China; 2 International School of Chinese Studies, Shaanxi Normal University, Xi’an, Shaanxi, China; East China Normal University, CHINA

## Abstract

**Background:**

Dialect Attitude is conceptualized as an individual’s cognitive and affective evaluation of a dialect and its speakers. In the contemporary China, dialect is suffering from significant stigmatization, resulting in social inequalities, which hinder sustainable development. This study aims to reveal the Chinese public attitudes towards dialects, and clarify the potential determinants related to heterogeneous attitudes at a macro level.

**Methods:**

We combine the crawler technology and sentiment analysis to conduct a provincial cross-sectional study. We collect 1,650,480 microblogs about public attitudes towards dialects from Microblog across 31 specific Chinese provinces. Spatial regression models are utilized to clarify the influence of macro-level determinants on differences in public attitudes.

**Results:**

The present study reveals that: (1) The Chinese public generally holds positive attitudes towards dialects, with significant variation between provinces. (2) Political Resource (β = 0.076, SD = 0.036, P<0.05), Economic Development (β = 0.047, SD = 0.022, P<0.05), and Cultural Resource (β = 0.054, SD = 0.021, P<0.05) promote public positive attitudes towards dialects. (3) Political Resource and Culture Resource influence more significant in the relatively advantaged regions, and Economic Development poses a higher influence in the relatively disadvantaged regions.

**Conclusions:**

Basing on the combination of crawler technology and sentiment analysis, the present study develops the most comprehensive database which takes 1,650,480 dialects-related microblogs from 31 Chinese provinces, and describes the following scenario: (1) Overall, the Chinese public shares a relatively positive attitude towards dialects with significant variations among different provinces, (2) Political Resource, Economic Development and Culture Resource pose positive effects on Chinese public attitudes towards dialects and (3) Political Resource and Culture Resource influence more significant in the relatively disadvantaged regions, and Economic Development poses a higher influence in the relatively advantaged regions.

## Introduction

Dialect Attitude (DA) refers to individual’s attitude towards a dialect and its users, which includes three specific emotional polarities, positive, neutral, or negative [1, 2]. According to the latest data from the China General Social Survey (CGSS), China is a multi-dialect country with more than one hundred dialects, and the dialect users have reached to 900 million, accounting for 64.69% of the total Chinese population [3]. Positive DA not only contributes to shaping the unique language variation of a nation, but also promotes social integration and sustainability [4, 5].

However, the desperate truth is that Chinese dialects are suffering from significant stigmatization, especially after the 2000 Chinese economic boom [6]. Chinese dialect users are associated with rude and vulgar, leading to a disadvantaged socioeconomic status and therefore are posed to the bottom of the social ladder [7, 8]. For example, the stigmatization of Andalusian dialects is the result of low socioeconomic status, low educational attainment and social isolation of dialect speakers [9].

Several established studies have shown that the stigmatization of dialects stems from three main factors: (1) language policies leading to divisions within dialect communities; (2) the perception of dialects as hindrances to economic development, and (3) dialects serving as markers of cultural differences that further deepen social divisions [10–13]. Unfortunately, the present literature on DA has primarily focused on micro-level factors such as income, education, gender and neglect the potential macro-level determinants correlates to DA [14–16]. Meanwhile, the limitation of sample size poses traditional linguistic analysis in a dilemma, which implies the need to combine big data with linguistics. To enrich scientific knowledge, this study collects dialects-related 1,650,480 microblogs from 31 Chinese provinces through crawling technique and sentiment analysis to investigate two crucial topics: (1) revealing the Chinese public attitudes towards dialects and (2) clarifying the potential determinants related to heterogeneous public attitudes.

## Theoretical framework and hypothesis development

There are four prominent academic theories beneficial for analyzing dialects, which including Systems Theory, Structuralist Theory, Planned Behavior Theory, and Social Network Theory [17–19]. Systems Theory offers a comprehensive framework to analyze DA, which emphasizing that the DA is embedded in the political, economic and cultural state of a given region [20]. Structuralist Theory posits that the use of dialects is not a personal choice, but is also closely linked to macro-level social characteristics [21]. Planned Behavior Theory offers an explanation and prediction of DA by incorporating subjective norms (social influence) and behavioral control (self-efficacy) [22].

These theories have been successfully applied in understanding public attitudes toward dialects, but each theory has it’s unique shortcoming in explaining DA. For example, Systems Theory suffers from imprecise boundaries that make testing specific hypotheses challenging [23]. Planned Behavior Theory emphasizes primarily individual-level factors and does not adequately address the influence of external structural factors on DA [24, 25]. Therefore, we employ the Social Network Theory from Brown as a fundamental theory, which has been proved to be useful for explaining the interaction between individual behavior and macro environment [26]. There are two main reasons for adopting this theory: (1) It posits that social networks could shape social capital that enables individuals to establish connections with the external world and gain social support, which finally shapes individuals’ behavior [27]. (2) It contends that political, economic, and cultural factors systematically and profoundly shape individual behavior, aligning with our proposed mechanism for influencing public attitudes towards dialects.

We develop a theoretical framework of the mechanisms influencing public attitudes toward dialects from the following three key perspectives of Social Network Theory as:

(1) Political Resource (PR), which is conceptualized as the political connections that individuals can access, either in material or immaterial form, to improve their socio-economic status [28]. PR affects DA through two heterogeneous paths [29]. On the one hand, PR positively influences DA. A harmonious society promotes inclusive social atmosphere and enhances positive public attitudes towards dialects [30–32]. For example, Social Assistance (SA) programs in developing countries effectively reduce dialect stigmatization [33, 34]. On the other hand, PR might pose a negative impact on DA. In a centralized polity, a disharmonious political environment undermines the sustainability of DA [35]. Some governments enforce official language and suppress dialect development, leading to a decline in DA. An extreme example is the political crackdown on Latin dialects by certain regimes in the United States [36].

In China, PR contributes to shaping positive public attitudes towards dialects, which are rooted in the unique political environment. Since 2010, the Chinese government has been committed to promoting an inclusive, harmonious policy support that endorse dialects as part of Chinese culture [37–39]. Therefore, we propose the first hypothesis.

*Hypothesis 1*: *Political resource positively influences dialect attitude*.

(2) Economic Development (ED), which refers exclusively to economic investment in cultural businesses or industries in this article [40]. The explanation path of the relationship between ED and DA is as follow: Firstly, a prosperous economy contributes to shaping an inclusive social climate, which in turn, enhances the sustainability of DA. A typical example is the economically developed Chinese province, Shanghai, where dialect-based economic groups are formed because of the favorable economic environment [41, 42]. Moreover, frequent trade and ED facilitate intercultural interactions and the spread of dialects [43, 44]. Secondly, strong social focus on ED could shape negative consequences for DA [45]. When economic efficiency becomes the sole priority, there is a risk of excluding or marginalizing certain dialect-speaking communities, as seen in the case of the Soviet Union [46].

In China, we propose that ED has a positive influence on DA. Because the Chinese government has placed the highest priority on ED since 1970, China has developed a mature ED system and a favorable social climate with social phenomena such as DA. Accordingly, we propose the second hypothesis.

*Hypothesis 2*: *Economic development positively influences dialect attitude*.

(3) Cultural Resource (CR), which is the availability of cultural support that contributes to an individual’s socioeconomic status, which can be either material form, such as cultural industry (CI) and cultural consumption (CC), or immaterial form, such as cultural transmission (CT) [47]. Extensive literature consistently predicts the significant relationship between CR and DA, where more inclusive social environments would support each individual richer social resources and accordingly foster the sustainability of dialects [48, 49]. CC encompasses activities such as the broadcasting of dialect programs and the publication of dialect literature, affecting the medium accessibility of individuals to the dialect [50]. Furthermore, CI represents the degree of economic support for culture by the government in a given country, which contributes to the economy and DA, simultaneously. Empirical research proves that Beijing, the most famous dialects city in China and characterized by Beijing speech, whose CI accounts for 15.12% of the total GDP [51].

Based on the above evidence, we propose that CR positively predicts the shaping mechanism of DA. Accordingly, we propose the third hypothesis.

*Hypothesis 3*: *Cultural resource positively influences dialect attitude*.

**Fig 1:**
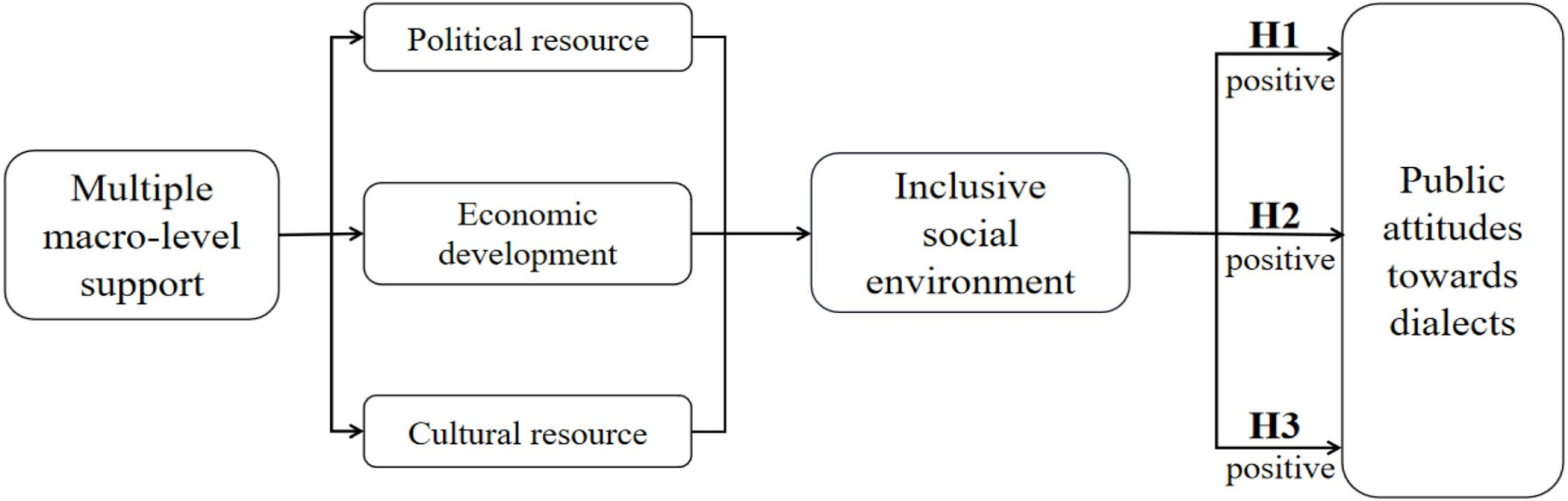
Analytical framework and potential mechanisms of the relationship between multiple macro-level support and public attitudes towards dialects.

Finally, we construct a comprehensive analytical framework and analyze the potential mechanism influencing DA in three macro factors: Political Resource, Economic Development, and Cultural Resource in Fig 1.

## Methodology

### Data

The present study uses Microblog as the data source, the most prevalent Chinese public media platform known for its anonymity and authenticity. During 2021/01/01-2022/10/01, we employed crawler technology to collect the tweets about dialects on the Microblog in each Chinese province. We collect three important information including user IDs, tweets contents and locations [52]. This technology proves particularly suitable for analyzing public attention on a given social phenomenon like DA, especially in a country like China where censorship regulations are stringent.

Therefore, we collect 1,690,932 related microblogs in all 31 Chinese provinces. Subsequently, we implement several filtering steps to ensure the integrity and quality of the data set as follow: we identify and remove microblogs that lacked provenance information (n = 26227), incomplete content (n = 14100) and duplicate entries (n = 125). Finally, we obtain a valid DA database including 1,650,480 microblogs from all 31 provinces in China in 2021/01/01-2022/10/01.

### Sentiment analysis

To ensure the suitability of the data for sentiment analysis, a crucial step is the pre-processing of the unstructured original data into structured data that can be analyzed. The data pre-processing stage involves three key steps:

1. Data selection. The initial step of data selection involves two interconnected word stemming processes. Stemming is employed to reduce word prototypes and their derived forms to a common basic form. This process involves the removal of prefixes, suffixes, and other inflections to obtain the root form of a word. For example, the derivatives ‘compute’, ‘computer’, ‘computing’, and ‘computed’ would all be transformed into ‘comput’ by discarding their distinct endings and retaining their shared components. Conversely, lemmatization is a more refined process that utilizes lexical and morphological analysis to reduce a word to its base form as it appeared in the dictionary. For instance, both ‘studies’ and ‘studi’ would be reduced to ‘study’. We perform the stemming and lemmatization using the ’Natural Language Toolkit’ (NLTK) package in Python.
2. Data cleaning. In the data cleaning stage, two primary operations are conducted to refine the data for sentiment analysis. Firstly, we convert all uppercase letters into lowercase letters. Secondly, we remove various punctuation marks, such as period (‘.’), comma (‘,’), question mark (‘?’), exclamation point (‘!’), and special characters including ampersand (‘&’) and slash (‘/’). Additionally, we eliminate all stop words, including ‘and’, ‘yes’ and ‘that’. The list of stop words is sourced from the public website (https://countwordsfree.com/stopwords).
3. Tokenization. Following data cleaning, the tokenization process is applied. Tokenization involves segmenting the documents into smaller units, typically at the word level. We employ the ‘split’ package in Python to accomplish this task.

**Fig 2:**
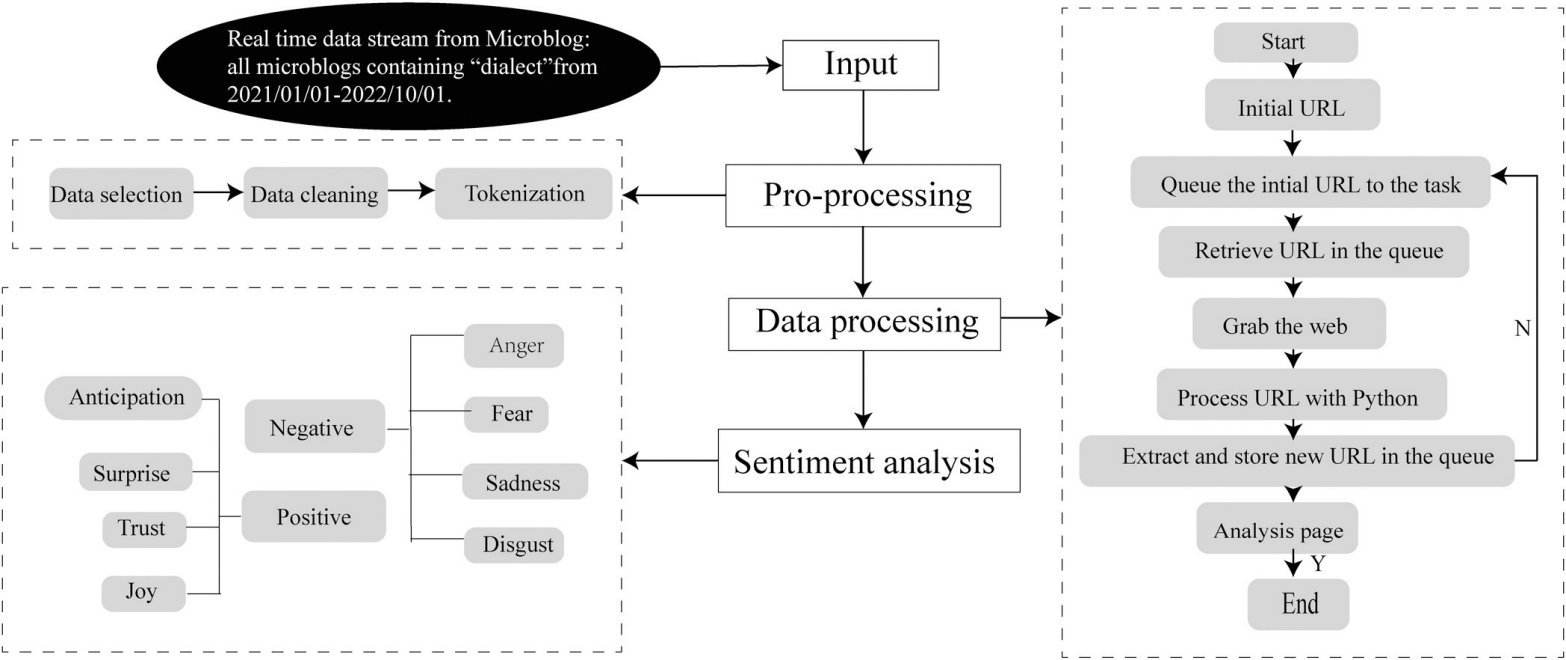
Principle and application of data collection.

Sentiment analysis is a methodology used to extract and analyze the opinions and attitudes expressed by social groups, with a focus on subjective emotional polarity, namely positive and negative sentiments [53, 54]. There are two main methods: machine learning-based sentiment analysis and sentiment dictionary-based sentiment analysis [55, 56]. Machine learning-based sentiment analysis is an important method to effectively determine Microblog users’ attitudes and sentiments towards dialects, but it requires a large amount of text training, which increases the cost of this study [57]. Compared with the former, sentiment dictionary-based sentiment analysis is more efficient and convenient because it uses an existing database [58, 59]. Therefore, we choose sentiment dictionary-based sentiment analysis. We utilize the sentiment dictionary developed by the National Research Council Canada (NRC), which encompasses a comprehensive range of language tags and eight emotional states, including anger, fear, sadness, disgust, anticipation, joy, trust, and surprise [60]. The first four of these are classified as negative emotions, while the last four are classified as positive. Sentiment analysis is performed using Python to assign sentiment scores to each word in the text. Finally, we sum up the scores of each dimension to obtain the total sentiment score for each microblog. The entire data pre-processing and sentiment analysis process are provided in Fig 2.

### Variables

#### Dependent variable

The Dependent variable is Dialect Attitude (DA). We use crawling techniques and sentiment analysis to obtain the percentage of microblogs with positive sentiment about dialect in 31 Chinese provinces. DA is a continuous variable ranging from 41.3% to 89.8%. In order to further analyze the variations in DA across different levels, we quintile this variable. Specifically, the range of 41.3% to 51.0% is categorized as “very low” (quintile 1), 51.1% to 60.7% as “relatively low” (quintile 2), 60.8% to 70.4% as “medium” (quintile 3), 70.5% to 80.1% as “relatively high” (quintile 4), and 80.2% to 89.8% as “very high” (quintile 5). The mean value of DA in the full sample is 3.06.

#### Independent variable

According to the theoretical model, DA is a function of PR, ED and CR. We select the independent variables from the above three perspectives, and all the data are taken from the National Statistical Yearbook (2022), available at http://www.stats.gov.cn/sj/ndsj/2022/indexch.htm.

1. Political Resource (PR). PR is an essential evaluation criterion that reflects political attention towards specific social phenomena such as DA. In order to assess PR, this article employs three commonly indicators, namely National Financial Allocation (NFA), SA and Employment [61, 62]. NFA is calculated by the central government’s financial allocation for dialect-related work in each province after counterbalancing the population and area, a continuous variable ranging from 14.80 to 174.31. SA is operationalized as the assistance by non-governmental organization for dialect work in each province, after counterbalancing the population and area, a continuous variable ranging from 4.00 to 373.30. Employment represents the employment rate for each given province, a continuous variable ranging from 19.30 to 703.90. We weight the three indicators to obtain a composite indicator, PR, which with the values ranging from 0.124–1.842. To more clearly distinguish the heterogeneous effect of PR on DA across different levels, we quintile this variable as follow: 0.124–0.468 is classified as a “very low” with value of 1, 0.467–0.812 is classified as “relatively low” with value of 2, 0.813–1.156 is classified as “medium” with value of 3, 1.157–1.500 is classified as “relatively high” with value of 4, and 1.501–1.842 is classified as “very high” with value of 5. The mean value of the PR in the full sample is 3.06.
2. Economic Development (ED). In order to capture the economic development situation, we access the three related economic variables: Gross Domestic Product (GDP), Consumer Price Indices (CPI), and Disposable Personal Income (DPI) [63, 64]. GDP is defined as the total economic output after counterbalancing the area and population. CPI represents the relative purchasing power of the necessities goods and services for the residents of a province, while DPI denotes the disposable income of its residents. All three indicators are continuous variables with specific value ranges: GDP ranges from 1902.74 to 110760.94, CPI ranges from 101.50 to 103.60, and DPI ranges from 21744.10 to 72232.40. To calculate the final ED indicator, all three variables are weighted, simultaneously. Consistent with the previous steps for defining variables, we quintile the ED, and obtain a five-category variable as follow: 1.129–1.984 is classified as “very low” (quintile 1), 1.985–2.839 is classified as “relatively low” (quintile 2), 2.840–3.694 is classified as “medium” (quintile 3), 3.695–4.549 is classified as “relatively high” (quintile 4), and 4.550–5.402 is classified as “very high” (quintile 5). The mean value of ED in the full sample is 3.12.
3. Cultural Resource (CR). We define the CR that incorporates insights from cultural sociology and history, which considering three key indicators: CT, CI, and CC. CT encompasses four sub-indicators, library, museum, radio, and television coverage, for a given province. CI represents the economic contribution of cultural industries in a given province, after counterbalancing the population and area. CC is calculated as the expenditure on culture and entertainment for a given province, after counterbalancing the population and area. All three indicators are continuous variables with CT ranging from 1.70 to 30.80, CI ranging from 3.20 to 992.50, and CC ranging from 1.80 to 539.60. We weight the three sub-variables and generate a new variable CR, which ranges from 0.330 to 5.116. Meanwhile, we quintile CR as follows: 0.330–1.287 is classified as “very low” with value of 1, 1.288–2.244 defined as “relatively low” with value of 2, 2.245–3.201 is “medium” with value of 3, 3.202–4.158 defined as “relatively high” with value of 4, 4.159–5.116 is “very high” with value of 5. The mean value of CR in the full sample is 3.06.

#### Control variables

The determinants of DA are highly intricate, and thus, in order to isolate the net estimation results, several control variables are considered, simultaneously. These variables include the gender (represent as a binary variable where 1 = male, 0 = female), years of education (indicate the number of years of education completed by respondents, ranging from 0 to 23) and the internet access rate (calculate as the proportion of individuals with internet access in a given province divided by the total population). All the data are taken from the National Statistical Yearbook (2022) (http://www.stats.gov.cn/sj/ndsj/2022/indexch.htm).

More details of the variables are given in Table 1.

**Table 1:** Variables description and operationalization.

Variables	Description	Operationalization	Source
DA	The percentage of microblogs with positive sentiment towards "dialect"	41.3% - 89.8%	Microblog
PR	The summary of political resource for each province	(NFA+SA+Employment)/3	CSY^a^
ED	The summary of the economic status for each province	(GDP+CPI+DPI)/3	CSY^a^
CR	The level of the cultural resource for each province	(CT+CI+CC)/3	CSY^a^
Gender	Gender of respondents	1 = male, 0 = female	CSY^a^
Years of education	Years of education completed by respondents	Years of education = (illiterate people*1 + people with elementary school education*6 + people with junior high school education*9 + people with high school and secondary school education *12 + people with college and bachelor’s degree or higher education *16) / Total population over 6 years old	CSY^a^
The internet access rate	The proportion of individuals with internet access in a given province divided by the total population	30.8%-47.9%	CSY^a^

Abbreviations: CSY, China Statistical Yearbook; NFA, National Financial Allocation; SA, Social Assistance; GDP, Gross Domestic Product; CPI,Consumer Price Indices; CT, Cultural Transmission; CI, Cultural Industry; CC, Cultural Consumption.

^a^ CSY available at http:/www.stats.gov.cn/tjsj/ndsj/2022/indexch.htm.

#### Spatial regression models

To estimate the mechanisms affecting cross-provincial heterogeneity in DA, we employ a Spatial Regression Model. The methodological reason for selecting this model over other models is that spatial autocorrelation resulting from the geographic location of individuals introduces significant bias in estimation, especially in the cross-provincial analysis. The first step is to verify that DA is spatially autocorrelated, as follows:

$$\left\{ \begin{array}{c} I_{i1}=\frac{\sum_{i=1}^{n}\sum_{j=1}^{n}w_{ij}(x_{i}-\bar{x})(x_{j}-\bar{x})}{S^{2}\sum_{i=1}^{n}\sum_{j=1}^{n}w_{ij}}\\ I_{i2}=\frac{\left(x_{i}-\bar{x}\right)}{S^{2}}\sum_{j=1}^{n}w_{ij} \end{array}\right.$$ (1)

The x in Eq (1) denotes the DA of a province. i and j denote the spatial weight array constructed based on the longitude and latitude of the province, respectively, and w_i, j_ represent the spatial weight matrix, which measure the spatial distance between region i and j. S^2^ is the sample variance. The upper part (I_i1_) is the global Moran index and the lower part (I_i2_) is the local Moran index. These indices are used to examine the spatial autocorrelation at a global and local level, respectively. The values of both sets of Moran indices range from -1 to 1. Negative values indicate the presence of negative spatial correlation, positive values indicate positive correlation, and values closer to 0 indicate a more random spatial distribution, suggesting that the spatial autocorrelation is not statistically significant.

After performing the Moran index test, this study examines the impact of the selected indicators on DA by employing two spatial econometric models, after balancing spatial endogeneity. The first model utilized is the Spatial Lag Model, as shown in Eq (2).


y=λWy+Xβ+ε (2)

In this model, X represents the matrix of independent variables that indicate the spatial distribution of PR, ED and CR across 31 provinces. Similarly, y represents the matrix of dependent variables that indicate the spatial distribution of DA across 31 provinces. W denotes the spatial weight matrix, λ denotes the spatial autoregressive coefficient, β denotes the matrix of parameters, *ε* denotes the random disturbance term.

The second model is the Spatial Error Model, as shown in Eq (3) below.


$$\left\{ \begin{array}{c} y=X\beta +\mu \\ \mu =\rho W\mu +\varepsilon \end{array}\right.$$ (3)

X is the matrix of independent variables, which indicate the spatial distribution of PR, ED, CR. y is the matrix of dependent variables, denoting the matrix of DA. β is the matrix of parameters to be estimated, W is the spatial distance matrix, μ is the spatial error term, and ε is the regression error term.

## Results

### Description statistics

**Fig 3:**
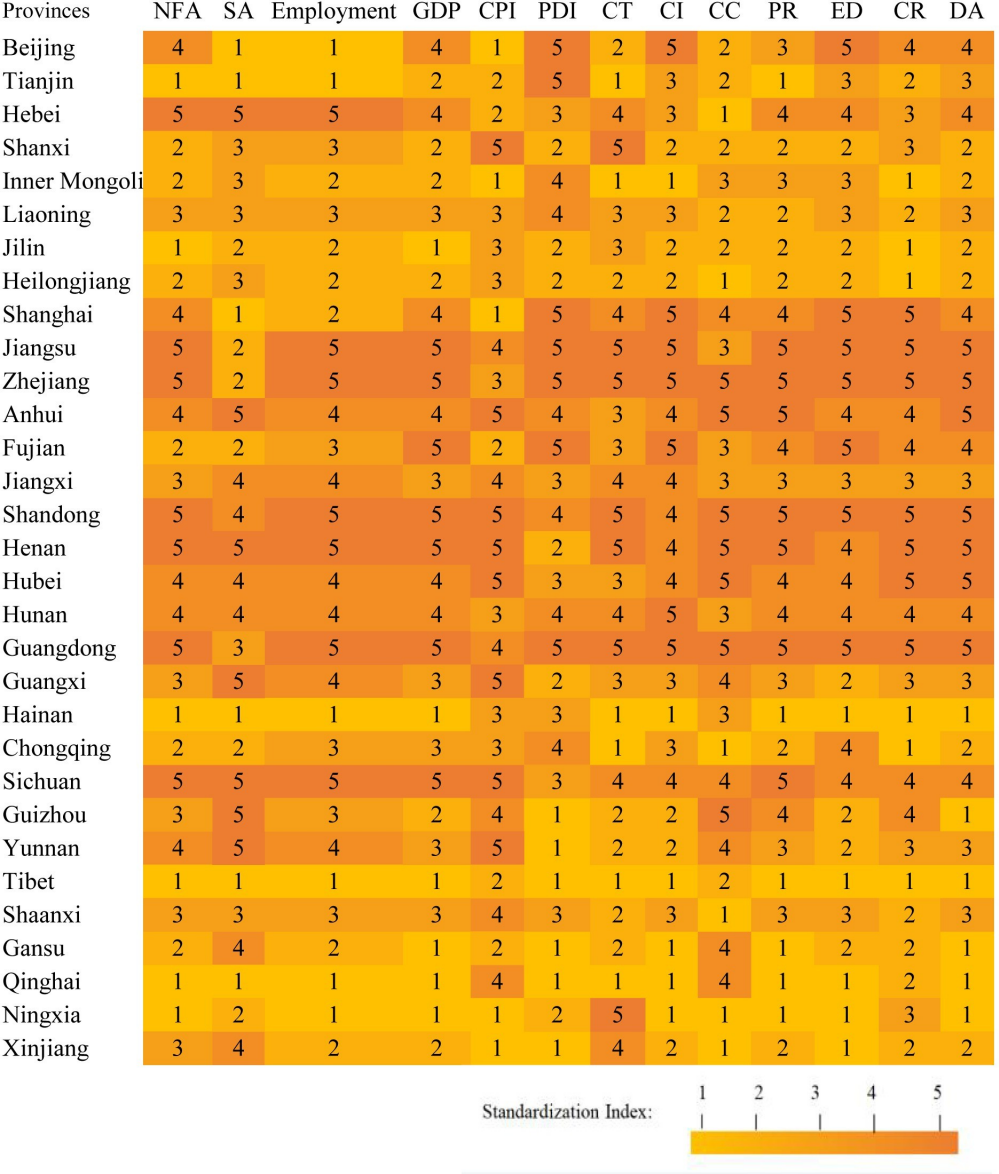
Descriptive statistics for 31 provinces in China (n = 1,650,480).

Fig 3 presents a comprehensive description of all indicators for the 31 provinces. Several meaningful preliminary findings are as follows: (1) Overall, the Chinese public shares a relatively positive attitude toward dialects, the mean value of DA is 3.06, which indicates that more than 60% of Chinese Internet users share a positive attitude towards dialects. However, it is important to highlight that there are significant variations in DA across different provinces. (2) The western region, characterized as the less-developed part of China, exhibits a significantly lower DA compared to the medium and eastern regions. Specifically, the mean of DA of the eastern, medium, and western regions are 3.91, 3.50, and 2.00, respectively. (3) Provinces with higher PR, ED and CR show significantly higher DA compared to provinces with lower indicators. Taken together, the data description captures that Chinese residents generally hold a positive perception of dialects, but there are significant regional differences that may be influenced by factors such as PR, ED, CR.

### Multicollinearity test

Because the selected variables in this study share the similar conceptualization processes and introduce potential multicollinearity problems which finally bias the estimation. Table 2 reports the correlation coefficients among the selected variables. Although some variables are correlated, the variance inflation factor (VIF) values of all variables range from 1 to 5 and are less than 10 [65]. Consequently, there is no significant multicollinearity.

**Table 2:** Pearson correlation analysis for all selected variables^a^.

Variables	1.	2.	3.	4.	5.	6.	7.
1.Gender	—						
2.Years of education	-0.089	—					
3.The internet access rate	0.236	0.231	—				
4.PR	0.011	0.127	-0.010	—			
5.ED	0.165	0.460**	0.304	0.755**	—		
6.CR	0.292	0.216	0.271	0.775**	0.907**	—	
7.DA	0.137	0.222	0.224	0.814**	0.901**	0913**	—

^a^ **p< 0.01.

### The determinants to Chinese public attitude towards dialects

#### Spatial auto-correlation test

**Fig 4:**
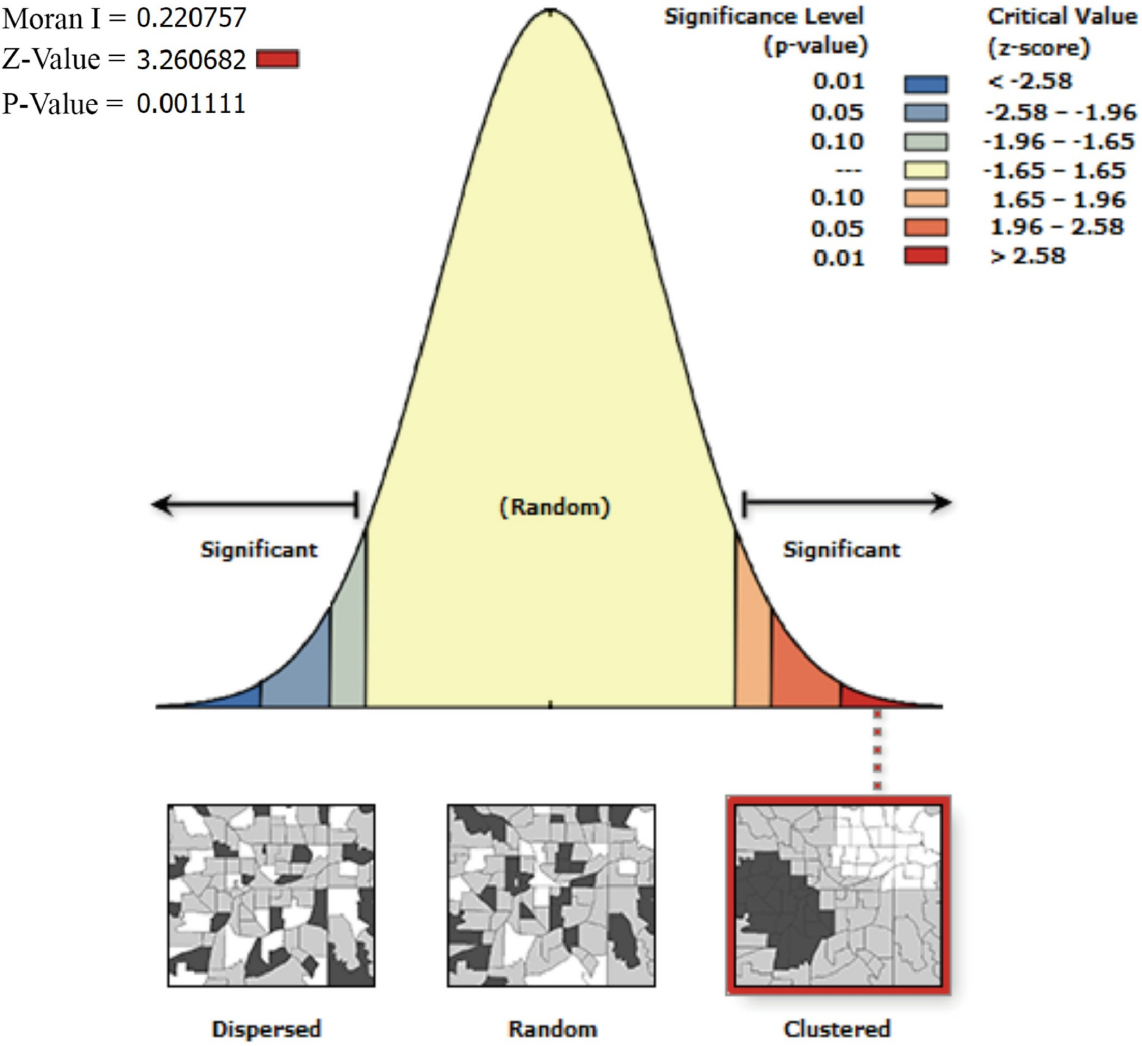
Global Moran index^ab^. ^a^ Moran_I_ > 0 indicates a positive spatial correlation of the attribute values in the region. ^b^ |Z|>1.96, p<0.05, Moran_I_ is significant.

Before conducting spatial regressions, it is essential to assess the spatial autocorrelation among the selected variables. Fig 4 illustrates the results of the global Moran index test, which reveals a statistically significant positive globally spatial-correlation (Moran_I_ = 0.221, Z-Value = 3.261), indicating that the spatial effects of DA in the provinces of China are distinct and exhibit geographic clustering.

Additionally, Table 3 provides local Moran index information of DA in China. It reveals that the local Moran index values are statistically significant in 8 of the 31 provinces, which indicates that the DAs in these provinces are clustered in their respective geographical locations. Taken together, these findings indicate that DA in China is significantly associated with geographical location. If this geographic bias due to spatial autocorrelation is not controlled, the estimations will be inaccurate. And accordingly, we will employ a spatial regression model to estimate the characteristics of DA.

**Table 3:** Local Moran index in 31 Chinese provinces.

Variables	Moran’s I^a^	SD	Z value	P value	Type^b^
Global Moran index:					
DA	0.221***	0.017	3.261	0.001	Significant
Local Moran index:					
Beijing	-0.042	0.102	-0.083	0.467	Not significant
Tianjin	0.096	0.105	1.227	0.110	Not significant
Hebei	0.028	0.106	0.580	0.281	Not significant
Shanxi	0.071	0.097	1.074	0.141	Not significant
Inner Mongoria	-0.009	0.084	0.292	0.385	Not significant
Liaoning	0.055	0.092	0.962	0.168	Not significant
Jilin	0.034	0.086	0.783	0.217	Not significant
Heilongjiang	0.008	0.075	0.553	0.290	Not significant
Shanghai	-0.297**	0.104	-2.532	0.006	H-H
Jiangsu	-0.773***	0.110	-6.739	0.000	H-H
Zhejiang	-0.444***	0.107	-3.818	0.000	H-H
Anhui	-0.363**	0.112	-2.940	0.002	H-H
Fujian	-0.250*	0.099	-2.173	0.015	H-H
Jiangxi	0.053**	0.110	2.605	0.005	L-H
Shandong	-0.338**	0.108	-2.810	0.002	Not significant
Henan	-0.409***	0.103	-3.660	0.000	H-H
Hubei	-0.307**	0.109	-2.518	0.006	H-H
Hunan	-0.174	0.106	-1.332	0.091	Not significant
Guangdong	-0.550***	0.096	-5.355	0.000	Not significant
Guangxi	0.082	0.094	1.236	0.108	Not significant
Hainan	0.116*	0.087	1.718	0.043	Not significant
Chongqing	0.046	0.093	0.850	0.198	Not significant
Sichuan	-0.001	0.086	0.375	0.354	Not significant
Guizhou	0.070	0.096	1.073	0.142	Not significant
Yunnan	0.005	0.085	0.446	0.328	Not significant
Tibet	-0.095	0.066	-0.944	0.173	Not significant
Shaanxi	0.054	0.088	0.994	0.160	Not significant
Gansu	-0.065	0.077	-0.404	0.343	Not significant
Qinghai	-0.114	0.069	-1.169	0.121	Not significant
Ningxia	-0.041	0.079	-0.099	0.461	Not significant
Xinjiang	-0.102	0.056	-1.225	0.110	Not significant

^a^ The surveyed provinces do not include Hong Kong, Macao and Taiwan

*p < 0.05

**p < 0.01

***p < 0.001.

^b^ The different types of spatial autocorrelation patterns are derived from the Local indicators of Spatial association (LISA) clustering map, where H-H = High-High Cluster, L-H = Low-High Cluster.

### Basic spatial regression estimation

Table 4 presents the empirical result of the basic regression model. Model 1 is a linear model using ordinary least squares (OLS), followed by Model 2, which employs Heckman model to address traditional endogenous problems. Finally, Model 3 utilizes structural equation modeling (SEM) to further improve the accuracy of the estimation, after balancing the bias caused by spatial autocorrelation. Panel 2 displays the model fitting information, and with the criteria of “comprehensive consideration of AIC and BIC information, the smaller the better”, we select Model 3 to report.

**Table 4:** The comparison between different selected model (OLS^a^, Heckman^b^, SEM^c^) of the correlation of PR, ED, CR on DA.

Variables	Model 1^d^ (OLS)	Model 2^d^ (Heckman)	Model 3^d^ (SEM)
Panel 1: Estimated coefficient.			
PR	0.095*** (0.001)	0.055 (0.039)	0.076* (0.036)
ED	0.037*** (<0.001)	0.113* (0.050)	0.047* (0.022)
CR	0.047*** (<0.001)	0.067** (0.023)	0.054* (0.021)
Panel 2: Model fitting of each selected models.			
AIC^e^	87.483	82.177	69.942
BIC^f^	81.747	73.265	57.239
AIC+BIC	169.230	155.442	127.181
AIC*BIC	7151.473	6020.698	4003.410
$\sqrt{{AIC}^{2}+{BIC}^{2}}$	119.732	110.095	90.378
CONS	0.385*** (0.030)	0.389*** (0.037)	0.303*** (0.064)
R2	0.8822	0.8824	0.8909

^a^ OLS denotes Ordinary Least Squares.

^b^ Heckman denotes heckman two-stage approach, a common means of balancing endogeneity problems.

^c^ SEM denotes spatial error model, which balance spatial endogeneity.

^d^ *p < 0.05, **p < 0.01, ***p < 0.001.

^e^ AIC refers to Akaike information criterion, the smaller the value the better the model.

^f^ BIC refers to Bayesian information criterion, the smaller the value the better the model.

The following meaningful conclusions regarding the potential determinants of DA of Chinese public are drawn: (1) PR poses a statistically significant positive effect on DA (β = 0.076, SD = 0.036, P<0.05). This implies that political intervention plays a crucial role in shaping positive public attitudes towards dialects, which aligns with previous research [66]. (2) ED has a positive statistically significant effect on DA (β = 0.047, SD = 0.022, P<0.05). This suggests that regional economic growth contributes to creating a harmonious social atmosphere and fostering positive DA. (3) CR poses a positive statistically significant effect on DA (β = 0.054, SD = 0.021, P<0.05). This finding is consistent with established literature, which emphasizes the importance of shaping an inclusive cultural atmosphere and promoting positive attitudes towards dialects [67]. In summary, the results confirm Hypothesis 1, 2, 3, respectively.

#### Heterogeneity analysis

**Fig 5:**
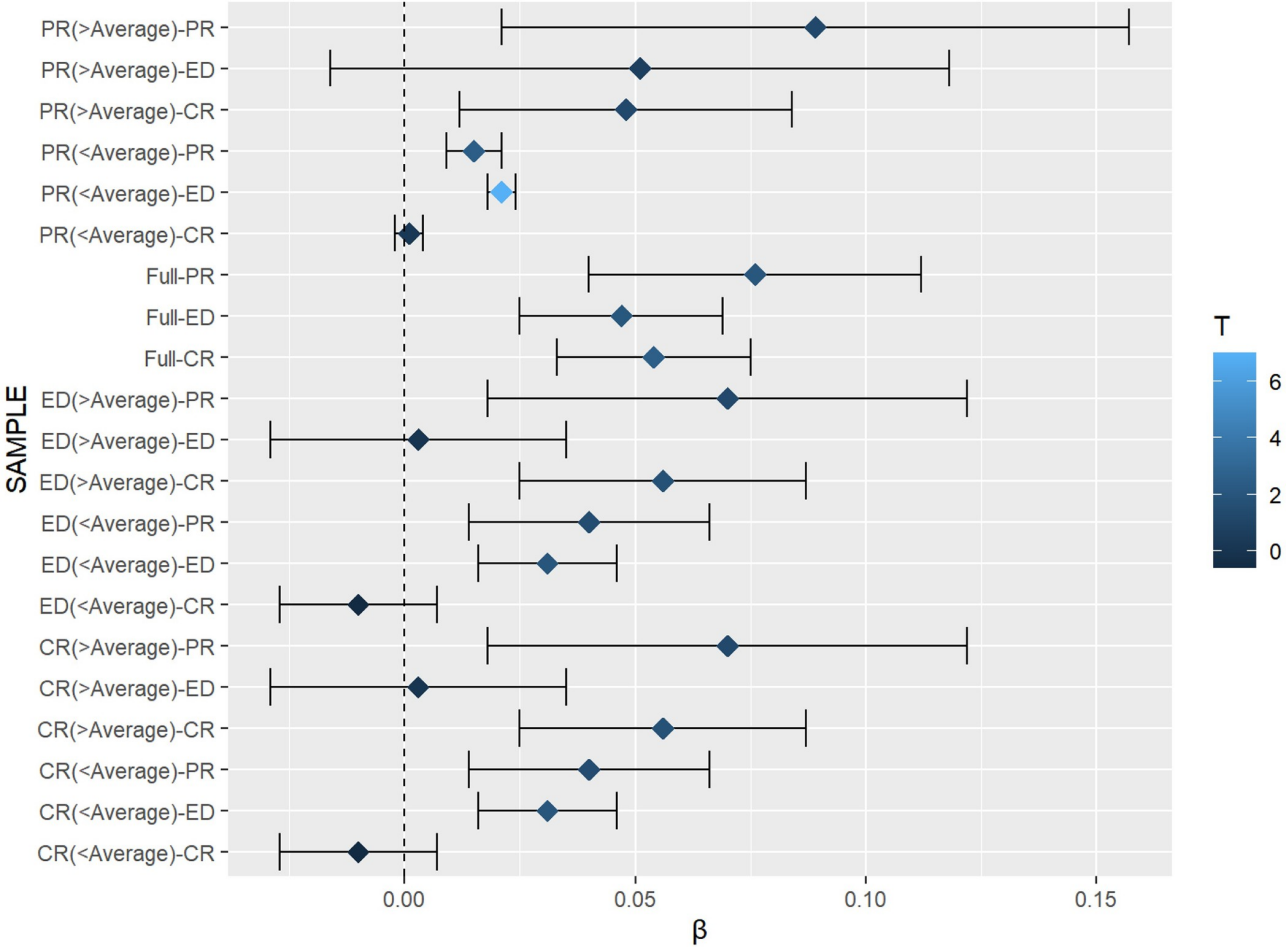
Heterogeneity analysis of different samples^ab^. ^a^ The closer the color is to black, the smaller the impact, and the closer it is to blue, the greater the impact. ^b^ T-value is significant at 5%, 1%, 1‰ at 1.65, 1.96, 2.76.

It is important to analysis the different influence across different samples, to draw more comprehensive conclusions across regions. Model 4–10 in Table 5 systematically present the results for high (above average) and low (below average) levels of PR, ED and CR. Some meaningful conclusions about the heterogeneous effects are drawn: (1) The positive effect of PR on DA is more pronounced in the relatively advantaged subgroups (higher PR, ED, and CR), compared to their disadvantaged counterparts. (2) The positive effect of CR on DA is more significant in the relatively advantaged subgroups (higher PR, ED, and CR), compared to their disadvantaged counterparts. (3) The positive effect of ED on DA is higher in relatively disadvantaged subgroups (lower PR, ED, and CR), compared to their advantaged counterparts. Taken together, political and cultural support have become more saturated in shaping positive public dialect attitudes, meaning that these two determinants are more likely to influence the advantaged counterparts. Meanwhile, economic support is still relatively scarce, so the positive influence of economic support remains at a marginal incremental benefit. The differences in the effects of different subgroups are visualized in Fig 5.

**Table 5:** Heterogeneity analysis of the influence across different samples^a^.

Variables	Model 4^b^	Model 5^b^	Model 6^b^	Model 7^b^	Model 8^b^	Model 9^b^	Model 10^b^
PR	0.076* (0.036)	0.089 (0.068)	0.015* (0.006)	0.070 (0.052)	0.040 (0.026)	0.070* (0.052)	0.038 (0.016)
ED	0.047* (0.022)	0.051 (0.037)	0.021*** (0.003)	0.003 (0.032)	0.031 (0.015)	0.004 (0.036)	0.031 (0.015)
CR	0.054* (0.021)	0.048 (0.036)	0.001 (0.003)	0.048* (0.027)	-0.010 (0.017)	0.056 (0.031)	-0.010 (0.027)
CONS	0.385*** (0.030)	0.380*** (0.078)	0.487*** (0.005)	0.574*** (0.079)	0.470*** (0.026)	0.574*** (0.079)	0.471*** (0.027)
R2	0.8822	0.7885	0.9174	0.7293	0.5326	0.7293	0.5328
Region	Full^c^	High PR^d^	Low PR^e^	High ED^f^	Low ED^g^	High CR^h^	Low CR^i^

^a^ We choose the optimal SEM reporting results.

^b^ * p < 0.05, **p < 0.01, ***p < 0.001.

^c^ Full denotes the whole group of subjects examined.

^d^ High PR indicates that the PR value is above the mean value of 24.242.

^e^ Low PR indicates that the PR value is below the mean value of 24.242.

^f^ High ED indicates that the ED value is above the mean value of 77.483.

^g^ Low ED indicates that the ED value is below the mean value of 77.483.

^h^ High CR indicates that the CR value is above the mean value of 49.790.

^i^ Low CR indicates that the CR value is below the mean value of 49.790.

#### Robustness

To test the robustness, we replace the sentimental dictionary, using Hownet dictionary and NTUSD dictionary. In addition, we replace the geographic distance matrix in the spatial regression with the economic distance matrix, and the results are shown in Table 6. It reveals that the robustness is maintained after replacing the dictionary and spatial matrix.

**Table 6:** Robustness test^a^.

Variables	Model 11	Model 12	Model 13	Model 14
PR	0.078* (0.024)	0.077* (0,034)	0.066* (0.033)	0.082* (0.037)
ED	0.037* (0.012)	0.039* (0.022)	0.042* (0.016)	0.053* (0.026)
CR	0.049* (0.019)	0.044* (0.011)	0.053* (0.018)	0.058* (0.029)
Dictionary	Hownet^b^	NTUSD^c^	Hownet	NTUSD
Spatial weights	Geographic	Geographic	Economic	Economic

^a^ * p < 0.05.

^b^ Hownet dictionary available at https://github.com/Shimon-Guo/chinese_sentiment_dictionary/tree/master/file/.

^c^ NTUSD dictionary available at http://nlg.csie.ntu.edu.tw/nlpresource/NTUSD-Fin/.

## Discussion

From the perspective of traditional academic views, political scientists, economists, and sociologists almost unanimously agree that dialects shaping social divisions and exacerbate social inequality [68, 69]. However, recent linguistic research suggests that dialects foster a more inclusive, sustainable, and cohesive social environment, and accordingly enhancing the well-being of local residents [14, 70]. To reveal the Chinese public attitudes towards dialects and clarify the potential determinants related to heterogeneous public attitudes, we use 1,650,480 microblogs collected from 31 Chinese provinces by crawler technology, and using sentiment analysis to analyze them. The findings of this study can be summarized as follows:

1. Overall, the Chinese public shares a relatively positive attitude toward dialects with significant variations across different provinces [71]. Fig 3 indicates that the mean value of DA is 3.06, suggesting that more than 60% Chinese public poses positive attitudes towards dialect. The positive attitude towards dialects observed among the Chinese public reflects the government’s efforts to preserve the various dialects [72]. Meanwhile, the present study also captures the significant variations of DA across provinces. Specifically, underdeveloped regions in the west part of the country exhibit lower DA compared to more developed medium and eastern regions. The above scenario indicates that there still exists systematically social stigma against dialects in Chinese disadvantaged regions [73]. Previous literature suggests that the promotion of the official language during China’s economic boom from 2000 to 2010 had a detrimental impact on dialects [74, 75]. Nevertheless, President Xi’s proposed dialect protection policies since 2015 till now have largely mitigated the crisis surrounding dialect [76].
2. PR, ED and CR pose positive effects on DA. Firstly, PR plays a significant positive role in shaping positive public attitude toward dialects (β = 0.076, SD = 0.036, P<0.05). The political support of protecting dialects is given the highest priority, especially in a country like China where the central government dominates [77]. For instance, political support has played a fundamental role in safeguarding Native American dialects in the United States, where they have been officially recognized as languages [78, 79]. Secondly, ED positive predicts the promotion of DA (β = 0.047, SD = 0.022, P<0.05). According to sociologists and economists, sociocultural phenomena such as dialects are embedded in economic development situation. In a developed economic region, the culture tends to be more inclusive, fostering the coexistence and development of multiple dialects rather than favoring a single official language [80]. Lastly, CR also contributes significantly to shaping positive public attitudes toward dialects (β = 0.054, SD = 0.021, P<0.05). Increasing cultural support for a region would imply the promotion of a more inclusive social climate, which is conducive to the development of dialects and has been confirmed in several established studies [81]. We extend it to China and find that cultural support remains an important part of efforts to safeguard dialects, despite the country’s predominant emphasis on economic advancement in the 21st century [82].
3. PR and CR influence more significant in the relatively advantaged regions, and ED poses a higher influence in the relatively disadvantaged regions. The results of heterogeneity analysis show regional heterogeneity in the effects of the selected factors on DA. Specifically, political and cultural support pose higher influence in developed regions, while economic support is more advantageous in underdeveloped regions. Taken together, political and cultural support result in diminishing marginal benefits, whereas economic support leads to incremental marginal benefits. This disparities could be attributed to the following scenario: Although Chinese economy booms, the government still under-invests economically in social culture (e.g., dialects). Instead, dialect development relies more on cultural and political support. Furthermore, different regions pose different political, economic and cultural situations. For instance, the most developed province have approximately 4.5 times higher GDP per capita compared to the least developed ones [83]. Meanwhile, underdeveloped regions are characterized by a greater diversity of dialects due to their more complex ethnic composition [84–86]. Therefore, it is important to implement region-specific strategies and shape positive public attitudes toward dialects [87, 88].

The study presents a comprehensive scenario on Chinese public attitudes towards dialects. And we propose several policy recommendations:

1. To ensure the sustainability of dialects, it is crucial to implement policies that focus on improving dialect quality and reducing regional inequalities. Drawing inspiration from successful European approaches, the Chinese government can consider adopting policies such as "multilingual parallelism policy" and "promote language attitudes policy" in Switzerland [89, 90]. These policies have demonstrated their effectiveness in addressing dialect inequalities and promoting positive DA.
2. Sufficient economic assistance plays a vital role in the protection of dialect, particularly in undeveloped regions. The government should allocate increased financial resources towards dialect protection initiatives, as evidenced by successful cases in other countries [91, 92]. Additionally, non-governmental organizations (NGOs) and enterprises should also assume the responsibility of safeguarding dialects by offering financial assistance [93].
3. Distinct strategies need to be implemented for different regions. In underdeveloped regions, it is imperative to cultivate positive public attitudes towards dialects through economic support. Conversely, in developed regions, the creation of an inclusive social environment through political and cultural support is necessary to foster public acknowledgement of dialects [94, 95].

## Conclusion, limitations and implications

Basing on the combination of crawler technology and sentiment analysis, the present study develops the most comprehensive database which takes 1,650,480 dialects-related microblogs from 31 Chinese provinces, and describes the following scenario: (1) Overall, the Chinese public shares a relatively positive attitude toward dialects with significant variations across different provinces, (2) PR, ED and CR pose positive effects on DA and (3) PR and CR influence more significant in the relatively advantaged regions, and ED poses a higher influence in the relatively disadvantaged regions.

The present study has several limitations to be explained carefully: (1) Data limitations. The findings of this study are based on provincial-level data, which may limit their generalizability to individuals-level attitudes. (2) Method limitations. The sentiment dictionary is not accurate enough, although it may work better using machine learning, this paper lacks a large number of texts to be trained. (3) Research aim limitations. This study focuses solely on exploring the macro-level mechanisms, which may overlook the potential influence of micro-level factors. (4) Endogeneity limitations. While this study addresses the endogeneity problem caused by geographic location, there may be other sources of endogeneity such as sample self-selection, omitting variables, reverse causation.

Despite its limitations, this study provides a comprehensive depiction of the Chinese public attitudes towards dialects and the macro determinants that influence these attitudes. And accordingly, the study makes three noteworthy implications for future study: (1) More big data-based sociolinguistic analysis is necessary, such as using Twitter to analyze public attitudes toward Native American accents in the United States and to reveal the social determinants. (2) Future research needs to incorporate a more integrated framework that takes political, economic, and cultural macro factors into consideration. (3) Sentiment analysis is an important research direction in sociolinguistics that needs attention [96].
